# Divalent Cu, Cd, and Pb Biosorption in Mixed Solvents

**DOI:** 10.1155/2009/561091

**Published:** 2009-08-13

**Authors:** M. H. Al-Qunaibit

**Affiliations:** Chemistry Department, King Saud University, P.O. Box 75318, Riyadh 11578, Saudi Arabia

## Abstract

Dead dried *Chlorella vulgaris* was studied in terms of its performance in binding divalent copper, cadmium, and lead ions from their aqueous or 50% v/v methanol, ethanol, and acetone solutions. The percentage uptake of cadmium ions exhibited a general decrease with decrease in dielectric constant values, while that of copper and lead ions showed a general decrease with increase in donor numbers. Uptake percentage becomes less sensitive to solvent properties the larger the atomic radius of the biosorbed ion, and uptake of copper was the most affected. FT-IR analyses revealed stability of the biomass in mixed solvents and a shift in vibrations of amide(I) and (II), carboxylate, glucose ring, and metal oxygen upon metal binding in all media. Δ_*ν*COO_ values (59–69 cm^−1^) confirmed bidentate metal coordination to carboxylate ligands. The value of *ν*_as_COO increased slightly upon Cu, Cd, and Pb biosorption from aqueous solutions indicating lowering of symmetry, while a general decrease was noticed in mixed solvents pointing to the opposite. M–O stretching frequencies increased unexpectedly with increase in atomic mass as a result of solvent effect on the nature of binding sites. Lowering polarity of the solvent permits variations in metal-alga bonds strengths; the smaller the metal ion, the more affected.

## 1. Introduction

Algal biomass represents an inexpensive material, that is, very efficient in heavy metal removal from effluents and industrial waters. Different mechanisms have been proposed to describe metal biosorption by algae where several functional groups are involved; carboxylates, hydroxyls, amines, phosphates, and sulfates (in marine species usually) are the most involved in metal binding [1–4]. *Chlorella vulgaris* (division *Chlorophyta*) usually does not contain sulfates [5].

According to Pearson [6] and complexation behavior of ligands and cations in terms of electron pair donating Lewis bases and electron pair accepting Lewis acids, metal ions are termed hard, soft, and borderline. Hardness of metal ions (Lewis acids) will determine their preference to binding. Softer ions (e.g., Cd^2+^, low positive charge relative to large size, very polarizable) are expected to bind nitrogen and sulfur donor atoms of the ligand on the algal cell wall, whereas hard metals (e.g., Ca^2+^, high charge to radius ratio, not very polarizable) coordinate to carboxylate groups, and borderline ions (e.g., Cu^2+^ and Pb^2+^) would bind to any of the ligands according to conditions that may change the hardness of the ligand. The same concept in hardness is used to describe solvents in terms of their abilities to bind ions [7]. 

 Solvent polarity is one of many factors affecting metal binding besides concentration, temperature, pH, and presence of competing species. Assessment of polarity scales employed bulk physical properties such as dielectric constant (relative permittivity) and other measures of chemical interactions such as donor numbers (important in coordination chemistry). The donor number (DN) [b] is a chemical measure (devised by Viktor Gutmann 1976) of Lewis basicity and is defined as the negative enthalpy value for the 1 : 1 adduct formation between a Lewis base and the standard Lewis acid antimony pentachloride, in dilute solution of the noncoordinating solvent 1,2-dichloroethane with a zero DN. The units are kilocalories per mole.

 Most of the studies on metal binding by algae were performed in aqueous media [1–8]. Biosorption investigations on other types of biomass, such as fungi [9] and natural products waste [10] followed the same methods of biosorption from aqueous solutions. Using solvents other than water in such experiments are almost exclusive of extraction purposes of materials adsorbed by algae [11] and in limited cases for metal ion adsorption capacity studies [8]. In an earlier study [12] ethanol-water Cu(II), Cd(II), Fe(III), and Sn(IV) solutions proved to enhance metal uptake by *Chlorella vulgaris* after extensive reuse of the biomass.

Using vibrational spectroscopy to study the cell wall of algae [3, 9, 10] and to investigate the metal coordination sites proved to be very useful in understanding the nature of metal-algae binding. To our knowledge, such studies on metal-loaded algae from mixed solvent solutions received no attention to date. Hence this study aims to investigate the biosorption of divalent copper, cadmium, and lead from different mixed solvents and the effect of the solvent properties on metal ion uptake and on metal-sensitive vibrations in the infrared region. 

## 2. Materials and Methods

The alga *Chlorella vulgaris* was generously provided by Dr. F. Al-Baz from the Botany Department at the National Research center, Cairo. The biomass was washed, air dried, ground, and sieved to a particle size of <355 *μ*m. Analytical grade nitrate salts of copper, cadmium, and lead were used as well as methanol, ethanol, and acetone of the same purity. Deionized water was used for all experiments. FT-IR spectra were recorded as KBr (10%) pellets using a Perkin-Elmer Spectrum 1000 FT-IR spectrometer. 

50% v/v mixed methanol/water, ethanol/water, and acetone/water were used in all experiments as mixed solvents. To determine the amount of the metal biosorbed by *Chlorella vulgaris*, 0.1 g biomass was added to 50 mL of 200 ppm metal solution, stirred for 10 minutes and left overnight, then filtered. Metal concentration was measured using ICP and the amount adsorbed calculated by difference. To prepare samples for IR measurements, 0.1 g of the biomass was added to 50 mL of 0.1 M metal solution, stirred for 10 minutes, and left overnight. Then filtered, washed, and dried at 80°C for one hour.

 The dielectric constant (*ε*_mix_) of each mixed solvent was calculated using the equation

(1) $$\varepsilon_{\text{mix}}=\frac{\varepsilon_{{\text{H}}_{\text{2}}\text{O}}V_{{\text{H}}_{\text{2}}\text{O}}+\varepsilon_{\text{solvent}}V_{\text{solvent}}}{V_{\text{total}}}$$

and presented in Table 1.

## 3. Results and Discussion

Values of dielectric constants of mixed solvents are all above 40, which reduce chances of formation of ion pairs in solution, and all ions are expected to be present as totally solvated in all solvents. In addition, polarizing ability of each of these ions is not enough to encourage ion pairs to form. Accordingly, metal ion species suggested are of the type [M(H_2_O)_6-n_(Sol)_n_]^2+^, a distorted octahedral moiety. Uptake from dilute solutions such as in this study renders precipitation on the algal cell wall unlikely to occur. 

The percentage uptake for each metal ion was calculated and presented in Table 1.

**Figure 1:**
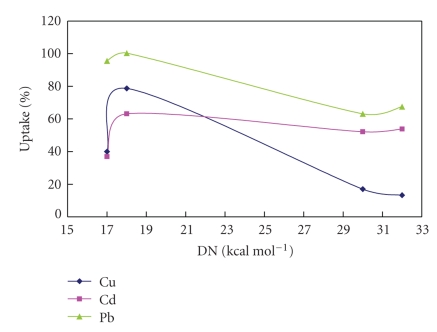
The effect of donor numbers on the percentage uptake of metal ions in different solvents.

Inspecting the values in the table above, it appears that the uptake percentage of the metal ions does not follow a clear trend in relation to the same property. But we can divide our observations into (1) cadmium ions showing a general decrease in percentage biosorbed with decrease in dielectric constant values (2) the harder copper and lead ions exhibiting a general increase in biosorption with decrease in donor numbers (Figure 1). The decrease from water to both alcohols is understood, as stronger forces between solvent and metal minimizes opportunities of competing ligands on algal cell wall. The unexpected higher uptake percentage from acetone solutions compared to alcohol solutions could be attributed to the fact that acetone is a nonprotogenic solvent which reduces chances for exchange with protons on the biomass. 

**Figure 2:**
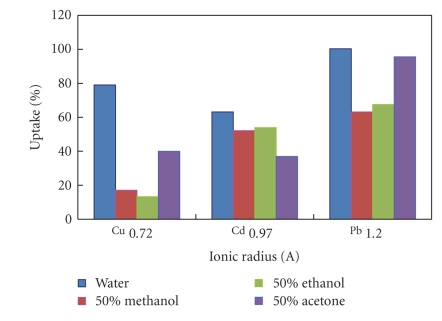
The effect of ionic radii on the percentage uptake in different solvents.

A plot of the percentage biosorbed versus ionic radii of the divalent metal ions (Figure 2) showed that, as the ionic radius increases from copper to lead, the uptake process becomes less sensitive toward changing the solvent.

Biological molecules such as algae show complex vibrational spectra that include overtones and combinational bands. But metal-ligand stretching frequencies and properties of functional groups coordinated to metal centers offer useful information. C–O stretching, NH_2_ rocking, and M–N and M–O stretching bands are metal sensitive and are shifted as the metal is changed, but NH_2_ vibrations are very sensitive to the effect of intermolecular interactions (e.g., hydrogen bonding) which makes it difficult to discuss the strength of the metal-nitrogen bond from the frequency shift. Doshi et al. [13] reported a blue shift of about 75–100 cm^−1^ of the band at 3304 cm^−1^ assigned to *ν*_NH_2__ coupled with hydrogen-bonded hydroxyl stretching in Spirulina sp. upon treatment with metal ions. Alcoholic groups in the glucose ring may play a role in metal binding, although Guibal et. al. [14] considered it constant and used it as an internal standard for calculating band intensities.

Assigning bands to the corresponding vibrations for biomaterials like algae is not a direct and easy task. According to Nakamoto [15], results of some researchers [1, 3, 14] and earlier work [16, 17], metal sensitive bands for the free biomass (water washed) are assigned as in Table 2. 

**Figure 3:**
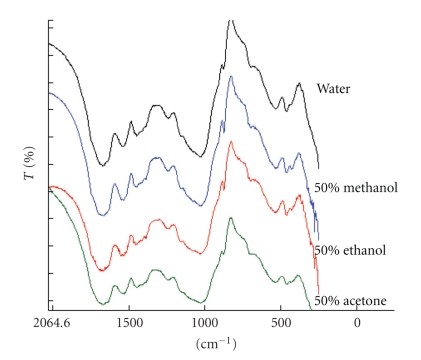
IR spectra of biomass in different solvents.

Mixed aqueous methanol, ethanol, or acetone did not seem to affect the functional groups responsible for metal binding in the biomass as can be seen from the IR spectra relative to water (Figure 3). 

Most FTIR studies on algae and seaweeds [1, 3, 14] and algal extracts [18] revealed the metal interaction sites of carboxyl, amino, and hydroxyl groups on the algal surface.

**Figure 4:**
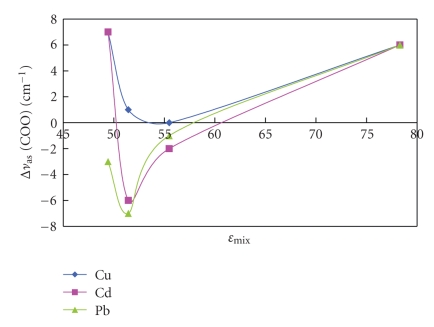
Change in Δ*ν*_as_COO with dielectric constant.

Introducing copper, cadmium, or lead into the biomass shifted all metal sensitive frequencies. For all metal loaded biomass samples, the separation (Δ_*ν*COO_) between *ν*_as_COO (asymmetrical stretching) and *ν*_s_COO (symmetrical stretching) was in the range of 59–69 cm^−1^, which conforms with bidentate coordination [15]. Hence, solvent polarity affected the amount adsorbed of the metal and accordingly shifted vibrations of metal sensitive bonds, but the mode of bonding was not affected. The shift in *ν*_as_COO (Δ*ν*_as_COO) varied upon metal binding and changing solvent, and a wider range of this shift was observed with decrease in dielectric constant values (Figure 4), where Δ*ν*_as_COO = (*ν*_as_COO free solvent − *ν*_as_COO metal-loaded biomass).

**Figure 5:**
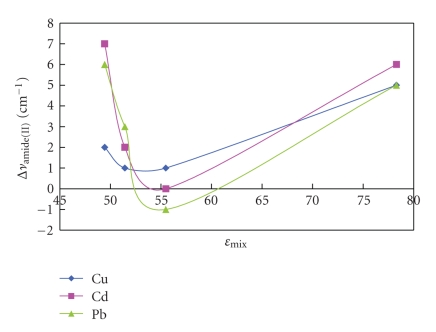
Change in amide(II) bands with e_mix_.

The positive shift in vibrations of samples prepared in aqueous solutions indicates lowering in symmetry for carboxylate coordination; on the contrary most samples from mixed solvents exhibited a negative shift presenting a more symmetrical environment for carboxylate. The same trend was observed in shifts of amide(II) bands in Figure 5. It can be concluded then that lowering polarity of the solvent permits variations in bond strength. 

**Figure 6:**
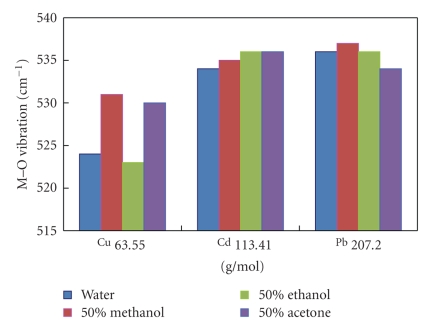
Change in M–O vibrations with atomic mass.

Figure 6 reveals an unexpected general trend; the stretching frequencies of M–O bonds increased with increasing atomic mass of the metal in all solvents. This may be a result of changes in the nature of the binding sites with changing solvents, which overcame the reduced mass effect. 

The last figure showed that copper biosorption varied in different solvents more than cadmium or lead did, which is what was noticed in Figure 2 (chemical analyses results). We can conclude then that the smaller the metal atom will be, the more affected biosorption will be by changing the solvent polarity.

## 4. Conclusions

Cadmium ions showed a general decrease in percentage uptake with decrease in dielectric constant values, while the harder copper and lead ions exhibited a general increase in biosorption with decrease in donor numbers. The metal ion uptake process becomes less sensitive toward changing the solvent the larger the atomic radius. 

Soaking dead dried *Chlorella vulgaris* in 50% v/v methanol-water, ethanol-water, or acetone-water did not alter the functional groups. Δ_*ν*COO_ for all metal loaded biomass samples was in the range of 59–69 cm^−1^ confirming bidentate coordination to carboxylate ligands. The value of *ν*_as_COO increased slightly upon Cu, Cd, and Pb biosorption from aqueous solvents indicating lowering of symmetry, while a general decrease was noticed from mixed solvents indicating the opposite. Lowering polarity of the solvent permits variations in bond strength. M–O stretching frequencies increased unexpectedly with increase in atomic mass of the metal in all solvents, a result—thought to be—because of the nature of binding sites being affected by solvents, making the reduced mass effect less pronounced.

## Figures and Tables

**Table 1:** Percentage of metal ion uptake from different mixed solvents.

Solvent	*ε*_mix_ [7]	DN [7]	%Cu	%Cd	%Pb
H_2_O	78	18	79	63	100
50% v/v methanol*	56	30	17	52	63
50% v/v ethanol*	51	32	13	54	68
50% v/v acetone*	49	17	40	37	96

*Whenever any of the 50% v/v mixed solvents is mentioned in the following, only the name of the solvent will be used.

**Table 2:** Metal sensitive IR vibrational frequencies of water washed C*hlorella vulgaris. *

Band	Group	Band (cm^−1^)
*ν*_C=O_, *δ*_N–H_	amide I	1665
*ν*_C–N_, *δ*_N–H_	amide II	1531
*ν*_as(C=O)_	Carboxylate	1446
*ν*_s(C–O)_	Carboxylate	1384
*ν*_C–N_, *δ*_N–H_	amide III	1240
*ν*_C–O_	glucose ring band	1030
	metal binding	876, 533, 462
